# The kinetics of amino acid disappearance in the small intestine is related to the extent of amino acids absorbed in growing pigs

**DOI:** 10.1017/S0007114523002441

**Published:** 2024-03-14

**Authors:** Carlos A. Montoya, Michael van Bemmel, Kevin Kreutz, Suzanne M. Hodgkinson, Natascha Stroebinger, Paul J. Moughan

**Affiliations:** 1 Smart Foods & Bioproducts, AgResearch, Te Ohu Rangahau Kai Facility, Palmerston North, New Zealand; 2 Riddet Institute, Massey University, Te Ohu Rangahau Kai Facility, Palmerston North, 4474, New Zealand

**Keywords:** Amino acids, Kinetics of absorption, Transit time, Pigs

## Abstract

This study evaluated the importance of a correction for amino acids (AA) released into the hindgut on a measure of AA absorption kinetics and tested whether AA absorption kinetics are related to the extent of AA absorption using the growing pig as a model for humans. Thirty-six nine-week-old pigs (22·3 kg) received a diet containing whey protein as the sole protein source for 8 d. Pigs received their last meal containing the indigestible marker titanium dioxide before being euthanised at 1, 2, 3, 4, 6 and 12 h post-feeding. The entire content of each gastrointestinal tract (GIT) region was collected to determine AA released into the hindgut, and the kinetics and extent of AA absorption (uncorrected and corrected for AA entering the hindgut). Amounts of AA released into the hindgut increased over time (e.g. 33 and 180 mg of Glu for 4 and 6 h post-feeding). The corrected apparent amount of each AA absorbed from the GIT lumen after 4 h post-feeding was generally lower (*P* ≤ 0·05) than the uncorrected counterpart. Differences in both the kinetics and extent of AA absorption were observed across AA. For example, the time to reach half of the apparent AA absorption (T50) was 1·5 and 3·4 h for Met and Arg, respectively, whereas their extent of apparent absorption was 93 and 73 %. Negative correlations between parameters related to kinetics and the extent of apparent absorption were observed (e.g. for T50 *r* = −0·81; *P* < 0·001). The kinetics of AA absorption is related to the extent of AA absorption.

The kinetics of amino acid (AA) absorption are important in relation to the effect of AA on whole-body protein metabolism^(1,2)^. For example, AA need to be delivered at specific times and amounts to the site of protein synthesis^(3,4)^. The kinetics of AA absorption are likely to be one of the contributing factors determining the extent of AA absorption in the small intestine, but this remains to be demonstrated. For example, the rate and extent of lysine absorption were lower than that for the methionine counterparts (8·4 %/h and 81 %, respectively, *v*. 9·7 %/h and 93 %) in rats fed a ^15^N labelled wheat/yeast diet^(5)^.

The kinetics of AA absorption are commonly studied by measuring plasma AA concentrations over time. This information could potentially be combined with that generated from ileal cannulated animals to understand the association between the rate of AA absorption and the extent of AA absorption. However, plasma concentrations are difficult to interpret as they are influenced by several factors such as splanchnic AA metabolism. In addition, the ileal cannulation approach allows for determining the extent of AA absorption to the end of the small intestine only. The ileal cannulation method does not allow determining the kinetics of AA absorption, as the test meal reaches the terminal ileum several hours post-feeding, which means that absorption values cannot be determined earlier. Furthermore, the digesta collected at the site of the cannula only represent material found at the terminal ileum, while other parts of the test meal are still transiting proximal gastrointestinal tract (GIT) regions.

An alternative classical approach is to use serial slaughter studies with animals to directly determine AA disappearance (assumed to equate with absorption) from the GIT lumen, by measuring total amounts of AA remaining in the GIT at different time points after ingestion of a meal^(6,7)^. Although this method is time-consuming, relatively expensive, requires the use of multiple animals and can only be applied with an animal model, it gives direct estimates of the kinetics of AA absorption in the same animal. In these types of studies, it is generally assumed that the absorption of an AA as such is complete by the end of the small intestine (ileum) and that AA that have disappeared from the GIT lumen have been absorbed. With this approach, AA released into the hindgut are not usually accounted for, despite that over time, unabsorbed AA do enter the hindgut. We hypothesise that the amounts of AA released into the hindgut are significant and need to be considered when determining apparent AA absorption with the latter serial slaughter approach. We also hypothesise that the kinetics of AA absorption are related to the extent of AA absorption.

This study aimed to demonstrate the effect of correcting for the AA released into the hindgut on measures of the kinetics of apparent AA absorption and to determine whether the kinetics and extent of apparent AA absorption are related. The study also allowed us to evaluate the use of a single dose of titanium dioxide (TiO_2_) as an indigestible marker under our experimental conditions. The growing pig was used as an animal model for adult humans^(8)^.

## Materials and methods

### Animals and housing

Ethics approval for the animal trial was obtained from the Animal Ethics Committee, Massey University, Palmerston North, New Zealand (application number 17/05). Entire male pigs (*n* 36, PIC Camborough 46 × PIC boar 356L, mean 22·3 (se 0·32) kg bodyweight) were obtained from a commercial farm. Pigs were housed individually in metabolism crates in a room maintained on average at 22°C with a 12 h/12 h light/dark cycle. Cages and toys were washed daily. Toys were rotated daily. During the feeding and cleaning, pigs were monitored for general health, alertness, dietary intake and scouring using a scoring system to determine whether pigs remained in the study. The endpoints to exclude pigs from the study were food intake, lethargy and diarrhoea.

### Diets and experimental design

Pigs were randomly allocated to each of the post-feeding time points, and some researchers and technical support staff were aware of this allocation. Pigs received a semi-synthetic diet for 8 d to ensure that they were adapted to the environment and to the diet. The adaptation diet contained wheat starch (521·2 g/kg DM diet), soyabean oil (140 g/kg DM), sucrose (100 g/kg DM), cellulose (40 g/kg DM), dicalcium phosphate (20·5 g/kg DM), vitamin and mineral premix (3 g/kg DM), salt (3 g/kg DM), the antioxidant Endox ® (0·3 g/kg DM) and whey protein isolate (172 g/kg DM; NZMP^TM^ Whey Protein Isolate 8855, Fonterra Co-Operative Group Ltd) as the sole protein source. To improve palatability, the diet was mixed with 400 ml of tomato soup (20 g Maggi-rich tomato soup mix (14 g carbohydrates, 0·5 g protein, 0·3 g fat), Nestlé New Zealand Limited). The diet fulfilled the nutrient requirements of the growing pig^(9)^. During the feeding period, pigs received two equal meals (2 % bodyweight/meal) at 08:00 and 17:00 h. However, on the final day of the feeding period, the last meal for each pig was provided at different times to ensure a 12 h fasting period on the following test day.

On day eight, pigs were deprived of water for 2 h before receiving their final morning test meal. The test meal did not include the vitamin and mineral supplements or the antioxidant contained in the adaptation diet^(10)^, as the test meal was also consumed by humans in a parallel but separate study. The indigestible marker TiO_2_ (1 g) was included in the test meal to allow the determination of its concentration in each GIT location. The test meal was given to the pigs after a 12 h fasting period, apart from a group of pigs (*n* 6) that were euthanised directly. The latter group of pigs received TiO_2_ in the last meal on day seven, and for the calculations, they were considered as being 12 h post-feeding of the last meal. The remaining pigs were anaesthetised 15 min before being euthanised at 1, 2, 3, 4 or 6 h post-feeding (*n* 6 pigs/post-feeding time). Considering the sampling time required for each pig (∼25 min), pigs received their last meal at 30 min intervals to ensure that they were euthanised at the assigned post-feeding time. In addition, the same number of animals per post-feeding time was euthanised in a day. The anaesthetic cocktail (0·12 ml/kg bodyweight of Zoletil 100 (50 mg/ml), Ketamine (50 mg/ml) and Xylazine (50 mg/ml); Provet) was administered by an intramuscular injection. Pigs were euthanised by an intracardiac injection of sodium pentobarbitone (0·3 ml/kg bodyweight of Pentobarb 300; Provet).

The body cavity was opened, and the stomach (at the oesophageal and pyloric sphincters), terminal ileum (at the ileocaecal valve) and the terminal rectum were isolated with clamps and the whole GIT was dissected out as described previously^(10)^. The stomach and the terminal ileum (last 30 cm before the ileocaecal junction) were then removed, and the remaining small intestine was uncoiled and divided into two equal lengths (proximal and distal small intestine, PSI and DSI). To ensure that all chyme and digesta were collected, the stomach and each small intestinal region were gently flushed several times with a saline solution (0·9 g NaCl/l). The caecum was removed, and the colon (including the rectum) uncoiled. Faeces excreted post-feeding were collected. The full caecum and colon were weighed, opened longitudinally, digesta were collected and caecal and colonic tissues were then washed, dried with paper towels and weighed again to determine total digesta on a wet basis. This was considered to be the most accurate way to determine total digesta in these GIT locations, as digesta adhere to these tissues. Caecal and colonic digesta, in contrast to small intestinal digesta, are less easily collected with flushing. Caecal and colonic (including faeces) digesta were thoroughly mixed before taking a representative weighed aliquot. Chyme and digesta (PSI, DSI, terminal ileal, caecal and colonic) were immediately frozen in dry ice, stored at –20°C, freeze-dried and ground. Chyme and small intestinal digesta samples (without terminal ileum) for each animal were pooled. The amounts of DM for the representative aliquots of caecal and colonic digesta were then used to calculate the total DM contents in the caecal and colonic digesta.

### Chemical analysis

The test meal, stomach chyme, digesta (PSI, DSI, terminal ileal, caecal and colonic) and food refusals were analysed for DM and TiO_2_^(11)^. The test meal, food refusals, pooled digesta and terminal ileal digesta were analysed for standard AA (using HCl hydrolysis, o-phthalaldehyde pre-column derivatisation followed by reversed-phase HPLC)^(12)^ and tryptophan (alkali hydrolysis)^(13)^. The test meal was also analysed for starch (Kit AA/AMG, Megazyme), crude protein (nitrogen × 6·25; using an elemental analyser LECO), total fat (using a Soxhlet apparatus and petroleum ether extraction) and total dietary fibre^(14)^.

### Calculations

The amount of each AA (AA_i_) in the diet, food refusals, pooled digesta and terminal ileal digesta at each post-feeding time were calculated as shown below with terminal ileal digesta as an example. The same calculations were used to determine the TiO_2_ content (g DM). The TiO_2_ content of the stomach, PSI and DSI were summed to determine the TiO_2_ content of the pooled sample.

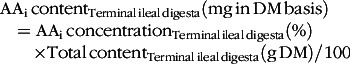




The relative amounts of DM and TiO_2_ exiting the stomach over time (time_i_ as 1, 2, 3, 4, 6 and 12 h post-feeding) were determined as follows (DM as an example):

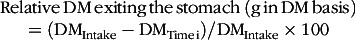




To ascertain the importance of AA escaping the small intestine into the large intestine, it was necessary to determine the amounts of AA released into the large intestine. Considering that the AA in large intestinal digesta do not represent the AA escaping the small intestine, as many are expected to be of microbial origin, the AA released into the large intestine at each post-feeding time were determined considering the sum of TiO_2_ content in the caecal and colonic digesta and the ratio of AA content/TiO_2_ content at the terminal ileal digesta (Direct method). Alternatively, the AA released into the large intestine can be calculated based on the TiO_2_ ingested in the meal and the TiO_2_ measured in the upper GIT (stomach to terminal ileum) (Indirect method). Both values were then used to determine the corrected apparent AA absorption in the GIT lumen as follows:

*Direct method*: AA_i_ released into the large intestine (mg in DM basis) = (TiO_2_ content_Caecal digesta_ + TiO_2_ content_Colonic digesta_) × AA_i_ content_Terminal ileal digesta_/TiO_2_ content_Terminal ileal digesta_

*Indirect method*: AA_i_ released into the large intestine (mg in DM basis) = (TiO_2_ content_Diet_ – (TiO_2_ content_Pooled digesta_ + TiO_2_ content_Terminal ileal digesta_)) × AA_i_ content_Terminal ileal digesta_/TiO_2_ content_Terminal ileal digesta_

Apparent AA_i_ unabsorbed (mg in DM basis) = AA_i pooled digesta_ + AA_i terminal ileal digesta_ + AA_i released into the large intestine_

Apparent AA_i_ absorbed (mg in DM basis) = AA_i_ content_Diet_ − (Apparent AA_i_ unabsorbed + AA content_Refusal_)

Apparent AA_i_ absorption (%) = (AA_i_content_Diet_ − (Apparent AA_i_ unabsorbed + AA content_refusal_)) / (AA_i_content_Diet_− AA content_refusal_) × 100

The mean food DM intake and DM in pooled digesta (stomach + PSI + DSI), terminal ileum and the large intestine as well as the lysine and TiO_2_ concentrations are shown in the Supplemental Materials and Methods to demonstrate the calculations described above. Data related to the study are available upon request.

One of the pigs at 4 h post-feeding did not have enough ileal digesta for both the AA and TiO_2_ analyses but enough contents in the remaining GIT locations. The TiO_2_ content of the remaining GIT locations was determined and 4 % of TiO_2_ reached the large intestine. To estimate the amount of AA released into the large intestine for this pig, a ratio between the average amount of AA released and the average amount of TiO_2_ in the large intestine for the same post-feeding time calculated over all the relevant pigs was calculated and multiplied by the amount of TiO_2_ in the large intestine for the specific animal.

### Statistical analysis

For the study, a sample size of six replicates per time point was deemed satisfactory (>80 % at a two-tailed 5 % significance level), based on an effect size of 1·86 obtained from data reported for the small intestinal AA digestibility^(15)^.

Statistical analyses were performed using SAS (SAS/STAT version 9.4; SAS Institute Inc.). A paired-*t*-test was performed to compare the determined and predicted amounts of TiO_2_ released into the large intestine. A polynomial analysis was conducted for the amounts of AA released into the large intestine. The best polynomial model (up to third order) for each response variable was selected after comparing higher- *v*. reduced-order models using the log-likelihood ratio test. Probability values of *P* ≤ 0·05 were considered of statistical difference, and 0·05 < *P* < 0·10 were considered a trend.

Non-linear models were fitted to the data, and the PROC NLIN of SAS was used to estimate the parameters of different models. To determine the transit time of the diet in each GIT location (stomach, PSI, DSI, terminal ileum, caecum and colon), the power exponential model (TiO_2_ remaining_Time_ = *α*_0_ exp – [*κ* × Time]^*β*^) was first used. *α*_0_ is the amount of TiO_2_ consumed (0·94 g), *β* is the index of the curve and κ is the slope of the curve. *β* and *κ* were then used to determine the 10 % cumulative transit time (CTT_10_ = [1/*κ*] × [log[1/0·9]]^[1/*β*]^). The time difference between cumulative transit times of different GIT locations was used to calculate the transit time of specific GIT locations (e.g. TT_10PSI_, h = CTT_10_ stomach and PSI – CTT_10_ stomach).

Based on the sigmoidal shape observed over time for the apparent relative AA absorption, different non-linear models (modified Weibull equation, Chapman–Richard equation, Logistic function and Gompertz function)^(16)^, commonly used for sigmoidal parameters, were firstly fitted for Asp, Glu and Leu. For these AA, the Gompertz function better fitted the data (online Supplementary Table 1). However, with the Gompertz model the point of inflection is not symmetric (i.e. the half time, T_50_ of apparent AA absorption cannot be determined). Thus, the Logistic function (relative absorption_Time_ = α/[1 + exp ^[*β* – *(γ*Time)]^]), which was the model with the second best fit (online Supplementary Table 1 and Supplementary Fig. 1), was fitted for all AA. The asymptote *α* represents the extent of apparent AA absorption or the apparent amount AA absorbed. The slope *γ*, or apparent absorption rate coefficient at *α*/2 (or inflection point), controls the shape of the curve and *β* shifts the curve along the X-axis. *β* and *γ* were then used to determine the point of inflection for *α* (T_α/2_ AA_i_ h = *β*/*γ*) and the time of 50 % for absorption of each AA (T_50_ AA_i_ h = *β*/*γ*).

The model diagnostics for each response variable were tested after combining the PROC UNIVARIATE and the ODS GRAPHICS procedures of SAS. A natural log or square root transformation of the AA released into the large intestine was required to fulfil the model assumptions of normality and homoscedasticity. The mean values reported are both transformed and back-transformed.

## Results

The chemical composition (g/kg DM diet) of the last meal consisted of 298 g of starch, 116 g of total fat, 48 of total dietary fibre and 214 g of crude protein. In the last meal, all animals, except three, consumed the whole meal in around 5 min. For the other animals, the refusals were removed after 15 min. Food refusals were generally low, and any remaining food from the last meal was weighed and food intakes were corrected. The mean intake of the test meal on the final experimental day was 203 (se 6·0) g.

**Fig. 1.**
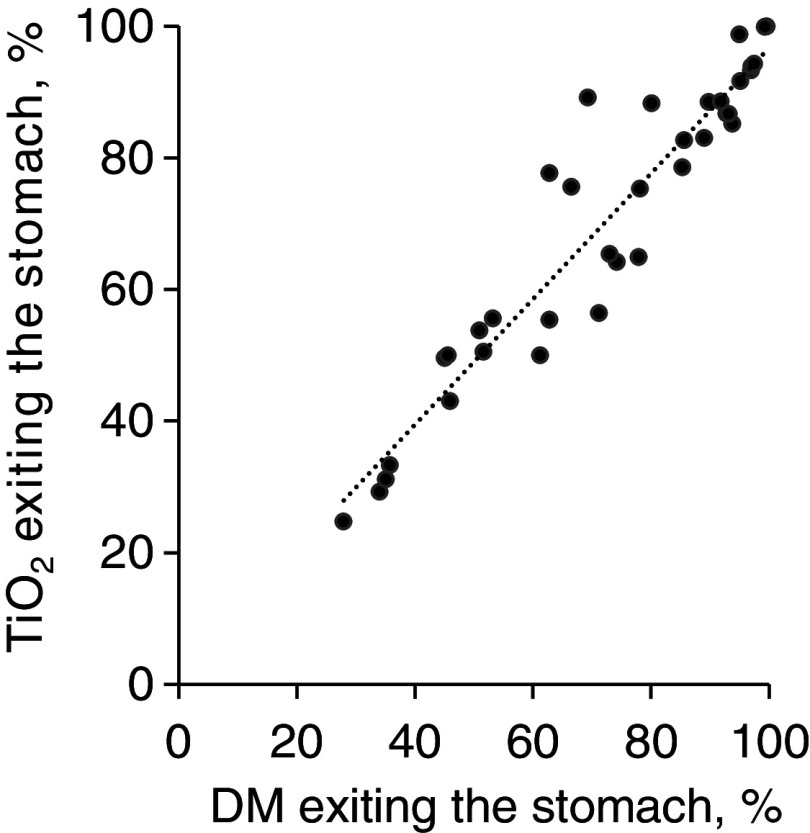
Correlation between the DM and titanium dioxide (TiO_2_) exiting the stomach over time for pigs fed a whey protein isolate containing test meal. The correlation value was 0·948 (*P* < 0·001, *n* 36).

**Fig. 2.**
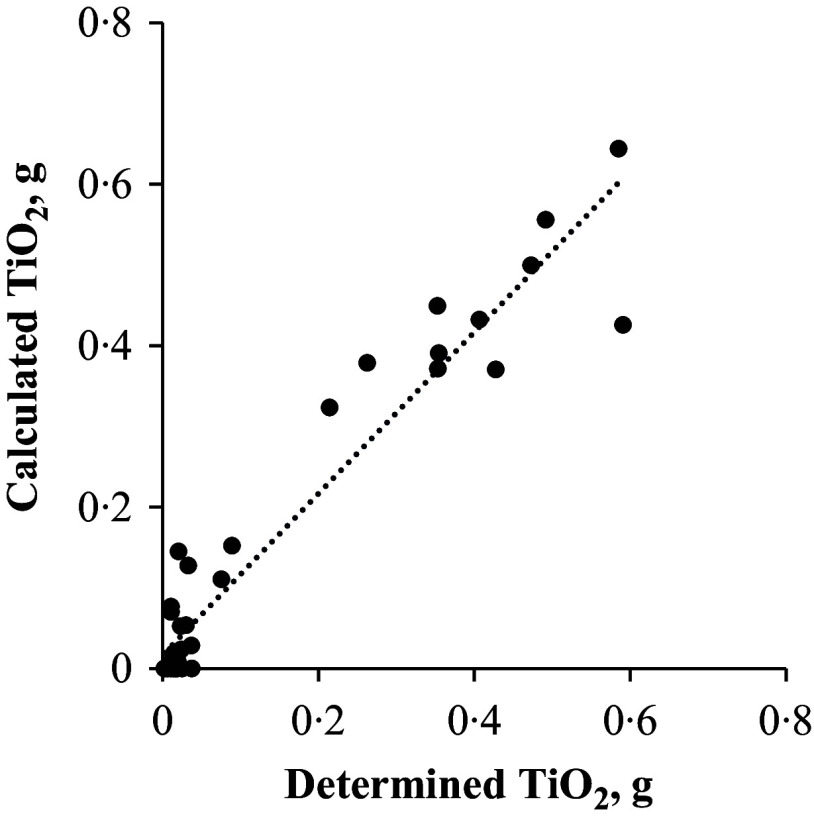
Correlation between the determined and calculated titanium dioxide (TiO_2_) content in the large intestine of pigs fed a whey protein isolate containing test meal. The correlation value was 0·96 (*P* < 0·001, *n* 36).

The mean recovery of TiO_2_ in the entire GIT and considering faeces excreted after consuming the test meal was 99·7 (se 1·1) %. The TiO_2_ exiting the stomach followed the same pattern (*r* = 0·948, *P* < 0·001) as the DM exiting the stomach (Fig. 1). There was a correlation (*r* = 0·96, *P* < 0·001; Fig. 2) between the amount of TiO_2_ determined and that predicted (calculated by the indirect method) to be present in the large intestine (Table 1). Only the results based on the direct approach are presented here.

**Table 1.** Determined and predicted amounts of titanium dioxide over time in the large intestine of pigs fed a whey protein isolate containing test meal* (Mean values with their standard errors)

Time, h	Determined		Predicted	
	Mean	SE	Mean	SE
	g			
1	0·013	0·006	0·006	0·085
2	0·025	0·008	0·013	0·062
3	0·013	0·005	0·011	0·036
4	0·037	0·026	0·038	0·068
6	0·313	0·130	0·352	0·123
12	0·452	0·145	0·473	0·117

* Values are means and standard errors of the mean, *n* 6 growing pigs per post-feeding time. Based on the paired *t* test results, the mean difference between determined and predicted titanium dioxide was not significantly different (*P* > 0·05).

Apart from one pig, AA were not released into the large intestine during the first 3 h post-feeding (Table 2). Therefore, the uncorrected apparent AA absorbed from the GIT lumen for the first 3 h post-feeding were the same (*P* > 0·05) as the apparent corrected values (Table 3). As expected, the amounts of AA released into the large intestine increased over time (*P* ≤ 0·05) from 4 to 12 h post-feeding (Table 2). For example, after back-transformation of the natural logarithm values (Table 2), the amount of Asp increased from 25 mg at 3 h post-feeding to 213 mg at 12 h. The corrected apparent amounts of AA absorbed from the GIT lumen after 4 h post-feeding were (or tended to be) lower (*P* ≤ 0·05) than the uncorrected counterpart. For example, at 6 h post-feeding the corrected apparent amount of Asp absorbed was 102 mg/g protein intake instead of 105 mg. Corrected values only were used to determine kinetic parameters for the apparent AA absorbed (mg/g protein intake) and apparent AA absorption (%).

**Table 2.** Amounts of amino acids released over time into the large intestine of growing pigs fed a whey protein isolate containing test meal* (Mean values with their standard errors)

	Post-feeding time, h												*P*
	1–3			4			6			12			
	Mean	SE		Mean	SE		Mean	SE		Mean	SE		
TiO_2_, %†	2·0	0·27		4·6	1·20		34	5·9		49	6·4		
*n* ‡	1			5			6			6			
mg													
His§	1·59	0·36	4·92	1·85	0·31	6·34	2·35	0·24	10·5	3·87	0·40	47·9	0·002L‖
Ile§	2·26	0·33	9·54	2·51	0·29	12·3	3·01	0·23	20·2	4·51	0·37	90·9	0·001L
Leu§	2·81	0·36	16·7	3·07	0·31	21·4	3·57	0·24	35·4	5·08	0·40	159	0·002L
Lys§	1·42	0·59	4·15	2·41	0·33	11·1	3·91	0·39	49·8	4·66	0·40	106	0·047Q‖
Met§	1·06	0·45	2·88	1·32	0·39	3·75	1·85	0·30	6·33	3·42	0·50	30·5	0·006L
Phe§	2·26	0·34	9·54	2·51	0·29	12·3	3·02	0·23	20·5	4·55	0·38	94·7	0·001L
Thr¶	4·63	1·23	21·4	5·55	1·07	30·8	7·38	0·83	54·4	12·9	1·38	165	0·001L
Trp§	1·71	0·35	5·52	1·95	0·30	7·01	2·43	0·24	11·3	3·86	0·39	47·5	0·003L
Val§	2·56	0·33	13·1	2·82	0·29	16·8	3·32	0·22	27·6	4·82	0·37	123	0·001L
Ala§	2·84	0·33	17·1	3·06	0·28	21·4	3·51	0·22	33·4	4·85	0·36	127	0·002L
Arg§	3·01	0·35	20·2	3·24	0·30	25·5	3·70	0·24	40·4	5·08	0·39	161	0·003L
Asp§	3·21	0·34	24·8	3·45	0·30	31·5	3·93	0·23	50·8	5·36	0·38	213	0·002L
Glu§	2·36	0·54	10·5	3·50	0·31	33·2	5·19	0·36	180	5·47	0·37	238	0·006Q
Gly¶	5·51	1·21	30·4	6·34	1·04	40·2	8·00	0·81	64·1	13·0	1·35	169	0·002L
Ser§	2·87	0·33	17·7	3·10	0·28	22·1	3·54	0·22	34·6	4·89	0·37	132	0·002L
Tyr§	2·23	0·35	9·28	2·46	0·30	11·7	2·94	0·24	18·8	4·35	0·39	77·8	0·002L
Total§,**	5·48	0·36	241	5·70	0·31	298	6·13	0·24	458	7·42	0·41	1,661	0·007L
EAA§,**	4·69	0·3	109	4·92	0·30	137	5·37	0·24	215	6·73	0·40	833	0·004L
NEAA§,**	4·94	0·36	139	5·16	0·32	174	5·60	0·25	270	6·92	0·40	112	0·005L

* Values are mean values with their standard error of the mean.

† TiO_2_, titanium dioxide_Large intestine_/titanium dioxide_Intake_ × 100.

‡ *n*, indicates the number of replicates. For post-feeding time 1–3 h, terminal ileal digesta of only one of the pigs at the 3 h post-feeding time had values higher than the limit of detection. Thus, for the animals with values lower than the limit of detection it was assumed that amino acids did not reach the terminal ileum and therefore they were not released into the large intestine. For post-feeding time 4 h, terminal ileal digesta collected were not sufficient to analyse titanium dioxide for one pig.

§ A natural logarithm transformation of the raw data was required to achieve the model assumptions of normality and homoscedasticity. The values (third column of the post-feeding time) represent the mean for each response variable after back-transformation.

‖ L or Q, linear or quadratic effect for the amounts of amino acids released into the large intestine over time.

¶ A square root transformation of the raw data was required to achieve the model assumptions of normality and homoscedasticity. The values (third column of the post-feeding time) represent the mean for each response variable after back-transformation.

** Total, EAA and NEAA, total, essential and non-essential amino acids.

**Table 3.** Uncorrected and corrected amounts of amino acids disappearing from the small intestine over time for pigs fed a whey protein isolate containing test meal* (Mean values with their standard errors)

	Post-feeding time, h†																			
	1		2		3				4				6				12			
	Mean	SE	Mean	SE	Mean	SE	Mean	SE	Mean	SE	Mean	SE	Mean	SE	Mean	SE	Mean	SE	Mean	SE
mg/g protein																				
His	2·58	1·63	5·38	0·37	10·4	0·6	10·4	0·6	12·2	1·0	12·1	1·1§	15·1	0·5	14·5	0·4*	16·0	0·2	15	0·5*
Ile	18·1	5·0	24·5	2·1	43·2	1·8	43·2	1·8	47·3	2·6	47·1	2·7*	54·3	1·3	53·2	1·2*	56·2	0·4	54·4	0·9*
Leu	47·6	11·5	65·9	4·9	104	4·3	104	4·3	117	5·4	116	5·4*	128	6·3	126	6·1*	137	0·6	133	1·6§
Lys	54·0	9·6	71·7	3·0	99·3	3·3	99·3	3·3	110	4·2	110	4·2*	126	1·3	124	1·2§	127	0·8	124	1·9§
Met	14·5	2·0	16·3	1·3	22·6	0·9	22·6	0·9	24·0	1·2	23·9	1·2*	27·0	0·5	26·4	0·5§	27·8	0·1	26·9	0·4§
Phe	9·69	3·24	15·1	1·1	27·4	1·2	27·4	1·2	29·8	2·3	29·5	2·3*	34·1	2·1	33·0	2·0*	36·9	0·4	35·0	0·9*
Thr	11·9	4·0	18·0	1·0	32·4	1·5	32·4	1·5	35·8	2·8	35·1	2·9*	43·5	1·0	41·8	0·8*	45·6	0·4	42·9	0·7*
Trp	13·1	1·8	16·2	0·7	22·2	0·9	22·2	0·9	24·8	1·0	24·7	1·0§	28·0	0·4	27·5	0·4§	29·1	0·1	28·3	0·4§
Val	13·9	3·9	19·9	1·4	33·8	1·5	33·8	1·5	37·2	2·5	36·8	2·5*	43·9	55	42·4	1·1*	45·2	0·7	42·9	1·2*
Ala	13·5	4·3	21·0	1·0	36·2	1·6	36·2	1·6	41·1	2·6	40·6	2·7§	47·2	2·9	45·6	2·7*	51·2	0·3	48·7	0·8*
Arg	4·1	1·69	6·73	0·67	14·6	0·8	14·5	0·8	16·1	1·9	15·3	2·1§	20·8	1·0	18·9	0·9*	22·0	0·4	18·6	1·1*
Asp	17·7	10·1	31·0	3·6	68·7	3·9	68·6	3·9	80·6	6·3	79·8	6·3*	105	2·0	102	1·6*	108	0·8	104	1·5*
Glu	28·6	14·5	52·2	5·4	102	5·5	102	5·5	123	7·2	122	7·3*	149	7·9	144	7·7*	164	0·9	159	2·2*
Gly	−9·91	2·27	−6·03	2·23	1·71	0·78	1·61	0·84	−0·10	4·90	−1·1	5·22*	7·11	1·53	5·2	1·5*	11·1	1·0	8·3	1·1*
Ser	4·18	3·44	10·0	1·0	21·5	1·5	21·4	1·5	24·0	3·2	23·4	3·3*	31·3	1·6	29·6	1·5*	33·7	0·4	31·3	0·8*
Tyr	7·93	2·81	11·7	1·3	22·5	1·1	22·4	1·1	24·1	2·3	23·8	1·2*	28·9	0·4	27·9	1·3*	31·0	0·4	29·3	0·8§
Total‡	247	65·1	379	22·7	663	29	662	30	746	47·9	739	49·0*	890	30·1	863	27·4*	942	7·38	902	16·1*
EAA‡	186	35·9	260	13·5	410	16·0	410	16·3	454	24·1	450	24·5*	521	13·7	508	12·1*	543	3·97	522	9·30*
NEAA‡	61·7	29·5	120	10·7	253	13·4	252	13·6	292	24·2	288	24·8*	368	16·5	354	15·4*	399	3·62	380	6·82*

* Values are mean values with their standard error of the mean, *n* 6 growing pigs per post-feeding time. Values (second means for post-feeding time 3 to 12 h) were corrected by amino acids released into the large intestine. Means with one asterisk differ (*P* ≤ 0·05).

† Corrected values are not reported for 1 and 2 h post-feeding times, as TiO_2_ did not reach the large intestine. Thus, uncorrected and corrected values are the same.

‡ Total, EAA and NEAA, total, essential and non-essential amino acids.

§ Mean values tended to differ (0·05 < *P* < 0·10).

The extent of the apparent AA absorbed (*α*) ranged from 15 mg/g protein for His to 154 mg/g protein for Glu (Table 4), whereas the apparent rate of absorbed AA (*γ*) at the inflection point (T_*α*/2_) ranged from 0·8 mg/g protein/h for Trp to 1·2 mg/g protein/h for Arg. The time to reach half of the total AA absorbed (T_50_) ranged from 1·6 to 3·6 h for Met and Arg, respectively, whereas T_*α*/2_ ranged from 1·2 to 3·0 h for Met and His, respectively. The observed and parameterised extent of apparent absorption for all analysed AA ranged from 73 to 95 % for Arg and Lys, respectively (Table 5). As expected, the parameters related to the rate of apparent absorption (e.g. *γ*, T_50_) were similar to the values for the apparent amounts of AA absorbed. For instance, the rate of apparent absorption ranged from 0·8 to 1·1 %/h for Trp and Arg, respectively, as reported above.

**Table 4.** Parameters of the Logistic function for the amounts of amino acids (mg/g protein) disappearing from the small intestine over time for pigs fed a whey protein isolate containing test meal*

	Parameters†									
	*α*, mg/g protein		*β*, h		*γ*, %/h		T_*α*/2_, h		T_50_, h	
	Mean	SE	Mean	SE	Mean	SE	Mean	SE	Mean	SE
His	14·8	0·59	2·72	0·44	1·12	0·18	2·97	0·14	3·10	0·12
Ile	54·2	1·86	1·98	0·32	1·02	0·16	1·95	0·10	2·29	0·11
Leu	131	4·46	1·76	0·30	0·98	0·15	1·81	0·10	2·14	0·11
Lys	125	3·48	1·39	0·22	0·89	0·12	1·55	0·07	2·10	0·11
Met	26·7	0·94	1·03	0·27	0·85	0·16	1·21	0·09	1·55	0·10
Phe	34·1	1·35	2·25	0·41	1·11	0·20	2·03	0·11	2·60	0·15
Thr	42·5	1·55	2·14	0·34	1·00	0·16	2·13	0·10	2·85	0·11
Trp	28·2	0·80	1·23	0·21	0·83	0·11	1·49	0·07	1·84	0·09
Val	42·9	1·53	1·97	0·33	1·01	0·16	1·95	0·10	2·49	0·13
Ala	47·6	1·73	2·12	0·34	1·02	0·16	2·08	0·10	2·62	0·14
Arg	18·7	0·94	2·70	0·58	1·19	0·26	2·28	0·14	3·56	0·25
Asp	103	3·8	2·82	0·40	1·08	0·16	2·61	0·11	2·97	0·12
Glu	154	5·8	2·56	0·37	1·02	0·15	2·53	0·11	2·99	0·13
Gly‡	–		–		–		–		–	
Ser	30·4	1·55	2·87	0·58	1·14	0·23	2·53	0·14	3·30	0·19
Tyr	28·7	1·25	2·24	0·42	1·05	0·19	2·14	0·12	2·73	0·16
Total§	890	30·5	2·09	0·31	0·98	0·14	2·14	0·10	2·49	0·11
EAA§	519	16·4	1·73	0·27	0·95	0·14	1·82	0·09	2·29	0·12
NEAA§	370	14·5	2·73	0·41	1·07	0·17	2·54	0·11	2·74	0·12

* The parameters of the model were estimated from amino acid disappearance corrected for the amino acid released into the large intestine.

† T_*α*/2_ is the point (time) at inflection. T_50_, the time at which half of each amino acid was apparently absorbed. T_*α*/2_ and T_50_ values were calculated based on fitting parameters (*β* and *γ*) of the Logistic function as detailed in Materials and Methods.

‡ The Logistic function did not converge for Gly.

§ Total, EAA and NEAA, total, essential and non-essential amino acids.

**Table 5.** Apparent amino acid disappearance from the small intestine over time for pigs fed a whey protein isolate containing test meal and parameters of the Logistic function for apparent amino acid disappearance* (Mean values with their standard errors)

	Post-feeding time, h												Parameters^†^									
	1		2		3		4		6		12		*α*, %		*β*, h		*γ*, %/h		T_*α*/2_, h		T_50_, h	
	Mean	SE	Mean	SE	Mean	SE	Mean	SE	Mean	SE	Mean	SE	Mean	SE	Mean	SE	Mean	SE	Mean	SE	Mean	SE
	%																					
His	13·9	7·84	31·1	2·17	60·4	3·72	70·3	6·15	84·6	2·49	87·7	2·62	86·1	3·37	2·79	0·43	1·15	0·18	2·43	0·11	2·88	0·13
Ile	30·4	6·96	41·7	3·59	73·4	3·12	80·0	4·54	90·4	1·99	92·5	1·51	92·2	3·13	1·95	0·30	1·01	0·15	1·94	0·10	2·19	0·11
Leu	33·5	6·65	46·6	3·44	73·8	3·04	82·1	3·84	89·3	4·29	94·4	1·14	92·8	3·12	1·73	0·28	0·96	0·14	1·80	0·09	2·04	0·10
Lys	39·0	6·29	54·5	2·25	75·5	2·51	83·6	3·16	94·5	0·87	94·5	1·45	94·8	2·60	1·44	0·21	0·91	0·12	1·58	0·07	1·75	0·08
Met	48·9	5·80	56·8	4·33	78·6	3·21	83·1	4·18	91·8	1·65	93·6	1·39	93·0	3·20	1·03	0·25	0·85	0·16	1·21	0·08	1·45	0·09
Phe	24·9	6·70	38·2	2·90	69·4	3·06	74·6	5·94	83·5	5·13	89·4	2·17	86·3	3·41	2·18	0·38	1·08	0·19	2·01	0·11	2·66	0·13
Thr	24·2	6·55	36·6	2·07	65·8	3·07	71·3	5·96	84·8	1·65	87·1	1·50	86·4	3·11	2·10	0·31	0·99	0·14	2·12	0·10	2·63	0·13
Trp	44·4	5·00	53·5	2·14	73·2	2·81	81·5	3·40	90·9	1·42	93·5	1·27	93·2	2·73	1·14	0·20	0·79	0·11	1·44	0·07	1·68	0·09
Val	26·2	6·96	40·7	2·89	69·1	3·14	75·3	5·20	86·8	2·18	87·9	2·55	87·6	3·09	2·06	0·32	1·04	0·16	1·98	0·10	2·41	0·12
Ala	25·7	6·45	38·7	1·77	66·7	3·01	74·8	5·00	84·0	5·01	89·8	1·50	87·8	3·20	2·04	0·31	0·99	0·15	2·07	0·10	2·51	0·13
Arg	17·4	5·52	26·0	2·58	56·1	3·18	59·1	8·29	73·2	3·62	72·0	4·43	72·5	3·69	2·53	0·52	1·12	0·23	2·26	0·14	3·44	0·24
Asp	15·6	7·24	27·3	3·16	60·3	3·46	70·2	5·55	89·6	1·43	91·3	1·33	91·0	3·31	2·80	0·37	1·07	0·15	2·53	0·11	2·89	0·12
Glu	16·4	6·91	30·4	3·13	59·4	3·22	70·8	4·26	83·9	4·46	92·4	1·27	89·3	3·28	2·57	0·35	1·02	0·14	2·64	0·09	3·01	0·10
Gly‡	−57·2	10·6	−33·1	12·2	8·81	4·61	−6·13	28·6	28·5	8·35	45·8	14·5	–		–		–		–		–	
Ser	12·5	7·67	26·9	2·72	57·5	3·97	58·1	7·99	79·7	4·06	84·0	2·09	81·8	4·13	2·79	0·53	1·10	0·22	2·52	0·14	3·20	0·18
Tyr	23·1	6·92	35·0	3·78	67·3	3·36	67·3	6·14	83·6	4·00	88·1	2·33	86·2	3·69	2·24	0·40	1·05	0·19	2·14	0·12	2·64	0·14
Total§	19·2	3·23	37·8	2·76	58·4	2·95	72·9	2·72	83·1	2·61	84·9	3·20	84·9	3·21	2·24	0·34	1·01	0·15	2·22	0·11	2·79	0·14
EAA§	26·6	3·11	46·6	2·45	65·8	2·66	78·2	2·33	87·0	2·36	88·7	2·91	88·7	2·92	1·80	0·27	0·95	0·14	1·89	0·09	2·30	0·11
NEAA§	8·77	3·32	23·2	3·89	45·8	3·85	64·4	3·86	76·5	3·41	77·9	4·00	77·9	4·00	3·28	0·64	1·21	0·25	2·71	0·14	3·54	0·19

* Values are mean values with their standard error of the mean, *n* 6 growing pigs per post-feeding time.

† T_*α*/2_ is the point (time) at inflection. T_50_, the time at which half of each amino acid was apparently absorbed. T_*α*/2_ and T_50_ were calculated based on fitting parameters (*β* and *γ*) of the Logistic function as detailed in Materials and Methods.

‡ The Logistic function did not converge for Gly.

§ Total, EAA and NEAA, total, essential and non-essential amino acids.

**Fig. 3.**
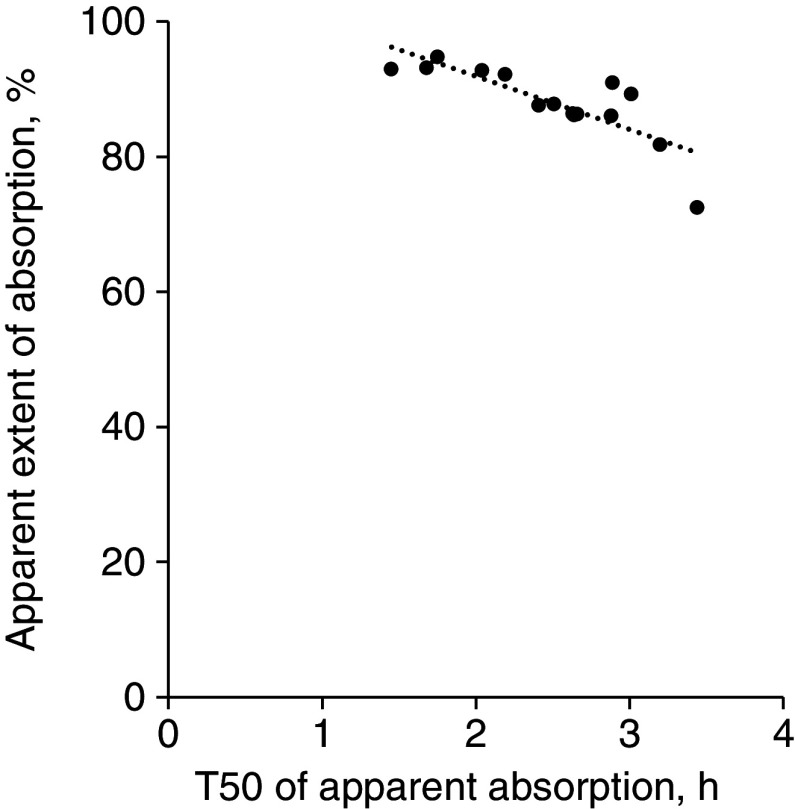
Correlation between the time at which half of each amino acid was apparently absorbed (T_50_) and apparent extent of amino acid absorption of pigs fed a whey protein isolate containing test meal. The correlation value was −0·81 (*P* < 0·001, *n* 15).

There were significant negative correlations between the apparent extent of AA absorption with both *γ* (*r* = −0·69, *P* < 0·01) and T_50_ (*r* = −0·81, *P* < 0·001; Fig. 3). The average T_50_ of the essential AA was shorter than the average T_50_ of the non-essential AA (2·19 *v*. 2·95 h, respectively; *P* < 0·01). Thus, essential AA tended (*P* = 0·06) to have a greater apparent extent of absorption than non-essential AA (90·3 *v*. 84·8 %, respectively).

**Fig. 4.**
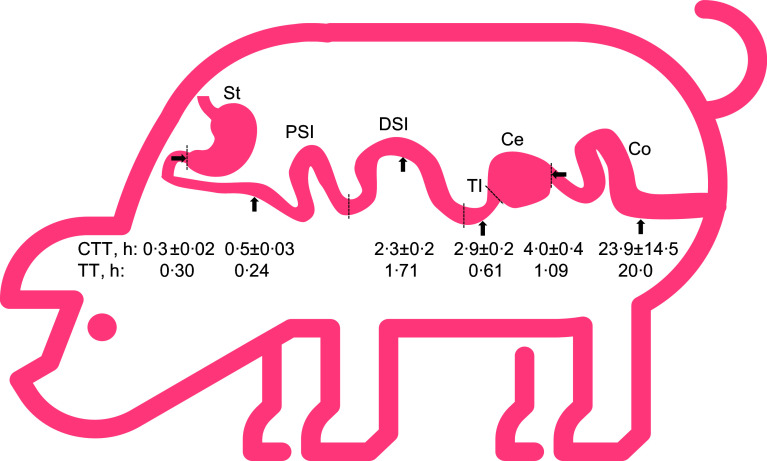
Cumulated transit time (CTT) and transit time at each GIT location (GIT, gastrointestinal tract; St, stomach; PSI, proximal small intestine; DSI, distal small intestine; TI, terminal ileum; Ce, caecum; Co, colon) for growing pigs fed a whey protein isolate containing test meal. The dotted lines represent the GIT location where the whole content was collected to measure the amount of the indigestible marker titanium dioxide, which was used to determine CTT and TT. Thus, the arrows represent the GIT location for both CTT and TT. Values are mean values with their standard errors, *n* 6. TT was calculated by subtracting CTT between GIT locations as detailed in Materials and Methods.

The amounts of TiO_2_ consumed and collected in each GIT location (stomach, PSI, DSI, terminal ileum, caecum and colon) were used to determine the 10 % transit time in each GIT location (i.e. the time required for 10 % of TiO_2_ to transit the given GIT location). The 10 % transit time for the stomach was 0·3 h (Fig. 4). The 10 % transit time of the diet from the mouth to the end of the small intestine (i.e., terminal ileum) was 2·9 h, while it was 4·0 and 24 h for the caecum and the mid-colon, respectively. In the small intestine, the 10 % transit time from the duodenum to the mid-PSI was 0·2 h, while from the end-PSI to the mid-DSI was 1·7 h. In the large intestine, the 10 % transit time in the caecum was 1 h, while from the beginning of the colon to the mid-colon it was 20 h.

## Discussion

One objective of this study was to evaluate the effect of the amount of AA released into the hindgut on measures of the kinetics and apparent extent of small intestinal AA absorption. A second objective was to test whether the kinetics of AA absorption are related to the extent of AA absorption. As hypothesised, there was a practically significant amount of AA released into the large intestine, which increased over time and needed to be considered to avoid overestimation of the kinetics of apparent AA absorption, when basing absorption on undigested material found in the upper digestive tract. Further, based on the correlation between several of the parameters related to the apparent kinetics of AA absorption and the apparent extent of AA absorption, the rate of AA absorption is strongly related to the extent of AA absorption.

### Amino acids released into the large intestine and corrected apparent absorption

Other studies have used portal vein cannulated animals to determine the kinetics of AA absorption^(17,18)^. However, as AA (e.g. Thr, Glu) are highly metabolised in the intestinal epithelium^(19)^, the portal vein AA flux only represents the transported AA. To determine the absorption of AA in the small intestine by mass balance, we needed to measure the AA remaining in the stomach, small intestine and those released into the large intestine over time. Here, we used TiO_2_ to track the flow of the test meal throughout the GIT and to determine AA released into the large intestine. Our results indicated that the amounts of AA released into the large intestine varied between AA (e.g. 3·8 and 40 mg of Met and Gly, respectively, at 4 h post-feeding) and increased over time (e.g. 33, 180 and 238 mg of Glu for 4, 6, and 12 h post-feeding).

Despite the modest differences between the uncorrected *v*. corrected apparent AA absorption values, the statistical analysis (paired-*t*-test) showed that for all AA, the uncorrected values overestimated or tended to overestimate apparent AA absorption. Considering that whey protein is a highly digestible protein, which means relatively low amounts of AA reach the large intestine, greater differences between corrected and uncorrected apparent AA absorption values are expected for more poorly digested protein sources. The differences found are likely to be of practical importance.

### Rate and extent of apparent amino acid absorption

Important differences in the extent of the apparent amount absorbed were observed across AA (15–154 mg/g protein). When those amounts were normalised for the AA composition of whey protein isolate, differences were also observed for the extent of apparent AA absorption (*α* values ranged from 73 to 95 %). Similar differences in the extent of apparent ileal digestibility (78–97 % for Gly and Met) have been reported in ileal cannulated growing pigs fed a raw beef-muscle protein-containing diet^(20)^. The wide difference in the apparent extent of absorption across AA could be explained by different factors such as the AA endogenous losses. For instance, the amount of the essential AA Thr was greater than other essential AA (670 *v*. <582 mg/kg DM intake) in ileal digesta collected from ileostomate adult humans fed a protein-free diet^(21)^.

Based on the reported literature, it was unknown whether the rate of AA absorption could also be one of the factors affecting the extent of absorption *in vivo* in a dietary protein, as previous studies of kinetics of AA absorption have been mainly done using *in vitro* and/or *ex vivo* techniques with purified AA^(22,23)^. Here, significant negative correlations were observed between variables related to the apparent rate of absorption (*γ* and T_50_) and apparent extent of AA absorption. These correlations were higher (e.g. for T_50_, *r* = −0·91, *P* < 0·001 *n* 9) when essential AA were only considered. Of the essential AA, Met, Trp and Lys had the shorter T_50_ and therefore the highest apparent extent of AA absorption, whereas His had the longer T_50_ and the lowest apparent extent of AA absorption. Previous studies using perfusion approaches in humans or rats have also shown that AA have different rates of absorption^(22,24)^. For instance, at 10 mM of perfusion in the jejunum of humans, Met had a greater absorption rate than Leu and Phe (129 *v*. 117 and 82 µM/min, respectively). The differences in absorption rates across AA observed here could be explained by competition for AA transporters^(23,25,26)^. However, there are several other factors (e.g. digestion, transit time) that could have affected the results observed here. For example, the faster small intestinal transit time of soyabean protein compared with casein^(27)^ could explain its lower portal-venous changes in rats^(28)^.

When the average values for T_50_ for essential and non-essential AA were compared, the essential AA appeared to be absorbed faster, which explains why they had on average a greater apparent extent of absorption (90·3 *v*. 84·8 %, respectively). The higher amount of non-essential AA released into the GIT lumen compared with the essential AA could partially explain the differences between the non-essential and essential AA. For instance, the average amount of the non-essential AA and essential AA reported here, apart from Trp, in ileal digesta collected from ileostomised adult humans fed a protein-free diet was 529 and 420 mg/kg DM intake, respectively^(20)^. Although it is unknown whether the same pattern of rates and extent of absorption between essential and non-essential AA remains for other dietary protein, it could be speculated based on the competition for AA transporters in the small intestine, and differences in AA composition across dietary proteins, that the relationship between parameters related to the rate of absorption and the extent of absorption differs across dietary proteins. Further work to relate the rates and extent of AA absorption across different dietary proteins for apparent and true values is warranted.

The appearance of essential AA in the peripheral blood of both young and older men given a whey protein meal peaked during the first 60 min postprandial^(1,29,30)^, but the results of this study showed that at 60 min less than one-third of the AA were absorbed. The appearance of plasma AA concentrations is difficult to interpret as they are influenced by several factors. Thus, further work to determine the correlation between absorbed AA and plasma AA concentrations is warranted.

### Transit time of the diet throughout the gastrointestinal tract

One of the main properties of a suitable indigestible marker is that it flows throughout the GIT simultaneously with the diet. In the present study, TiO_2_ exhibited similar stomach emptying to the chyme. Once the TiO_2_ has reached the small intestine, it is assumed that TiO_2_ flows with the digesta through the remaining GIT. For instance, a high correlation (*r* = 0·99, *n* 3) was observed between the average amount of TiO_2_ and the average amount of DSI digesta between 3 and 6 h post-feeding. The first 2 h and 12 h post-feeding times were not considered here, as 10 % DSI transit time occurs past 2 h (Fig. 4), and accumulation of digesta in humans at the terminal ileum has been reported at 6 h using a scintigraphy approach^(31)^. The 10 % stomach emptying time, and 10 % small intestinal and caecal transit times were 0·3, 2·9 and 4·0 h, respectively. In humans fed a 50 g test meal (scrambled eggs and two slices of white bread) and 150 ml water, the comparable 10 % transit times of the diet were on average 0·3, 4·7 and 5·0 h, respectively^(31)^. Assuming that the small intestine of the growing pig is on average 15 m long, the digesta moved from the duodenum to the terminal ileum at 10·4 min/m. However, when considering individual locations (i.e. dotted lines and arrows in Fig. 4), the rate of transit during the first quarter of the small intestine was faster (3·8 min/m) than for the third quarter (27 min/m). Using scintigraphy imaging and using the hepatic flexure of the colon as starting point for the left upper quadrant (considered as PSI) and right lower quadrant (DSI), faster transit time in the PSI compared with the DSI (57 and >390 min of half transit time) has also been reported in humans fed pancakes containing 15 g bran^(32)^. In pigs fed diets containing different starch sources, it has been shown a shorter mean retention time in the PSI compared with the DSI^(33)^. Surprisingly, 30 % of the intake of TiO_2_ was present at the DSI after 12 h post-feeding (data not shown), which suggests that an important amount of the undigested diet remained at the DSI (20 g) for an extended period of time. Such accumulation has also been reported at the ileum of humans^(31,34)^ and rats^(6,35,36)^ but has not been quantified.

### Direct *v*. indirect determination of amino acids entering the large intestine

To determine the amounts of AA released into the large intestine directly, total collection of caecal and colonic digesta is required to allow determination of the amount of TiO_2_ in the large intestine. However, unrolling the colon and collecting total caecal and colonic digesta are difficult and time-consuming. In this study, the amount of TiO_2_ released into the large intestine was calculated after unrolling the colon and with an almost total collection of caecal and colonic digesta (i.e., direct method) but also by subtracting the amount of TiO_2_ found in the stomach to the terminal ileum, from the amount of TiO_2_ ingested (i.e. indirect method). The statistically significant high correlation between TiO_2_ released into the large intestine using the direct and indirect methods suggests that the indirect method could be used to calculate the amounts of AA released into the small intestine. A limitation, especially with the indirect method, however, is that the terminal ileum does not always contain digesta, which affects the calculation of TiO_2_ released into the large intestine. The direct method allows to determine the exact amount of TiO_2_ in the large intestine, and this information can be used to calculate the AA released into the large intestine. Nevertheless, the indirect method appears to be a satisfactory alternative.

TiO_2_ reached the large intestine during the first 3 h post-feeding in small amounts, but when determining the TiO_2_ concentration in the ileal digesta only one of the pigs at the 3 h post-feeding time had values higher than the limit of detection (0·083 mg/ml TiO_2_). Thus, for all the pigs within the first 3 h post-feeding, except for the one pig, it was assumed that AA were not released into the large intestine (i.e. all the food AA were either absorbed or found within the pooled luminal contents). It is important to mention that AA were measured in the terminal ileal digesta of those animals, and these AA, which could be from both dietary (previous meals) and endogenous origin, are released into the large intestine but may not have been part of the test meal.

### Conclusions

There are quantitatively important amounts of AA released into the large intestine during digestion, which increase over time and need to be considered to avoid overestimating apparent AA absorption values with the serial slaughter total GIT content recovery method. The practical significance of the amounts of AA lost to the large intestine is expected to be greater for less digestible proteins. High negative correlations between the kinetics of AA absorption and the extent of AA absorption were observed when growing pigs were fed whey protein isolate as the sole protein source. This suggests that the kinetics of AA absorption modulates the extent of AA absorption. Based on the determined kinetics of AA absorption, essential AA are absorbed faster than non-essential AA. The absorption kinetics combined with transit time appear to modulate the overall extent of absorption of each AA.

## Supporting information

Montoya et al. supplementary material Montoya et al. supplementary material

## Supplementary material

For supplementary material referred to in this article, please visit https://doi.org/10.1017/S0007114523002441
