# Supplementary material for: The kinetics of amino acid disappearance in the small intestine is related to the extent of amino acids absorbed in growing pigs
Source: Br J Nutr. 2023 Oct 26;131(5):762–72. doi: 10.1017/S0007114523002441 (PMC10864992; doi:10.1017/S0007114523002441)
Supplement: Montoya et al. supplementary material [file S0007114523002441sup001.docx]

**Supplementary Methods**

Values for 6 h post-feeding are shown below to exemplify the calculations. The mean DM intake and DM in pooled digesta, terminal ileum, and the large intestine were 213, 31.6, 1.97, and 240 g respectively. The TiO_2_ concentrations were 0.43, 1.55, 4.15, and 0.14% respectively, while the lysine concentrations (except large intestine) were 2.86, 7.74, and 8.60% respectively.

Lysine intake _Pooled digesta_ (g) = 2.86% x 213 g / 100% **= 6.09 g**

Lysine content _Pooled digesta_ (g) = 0.774% x 31.6 g / 100% **= 0.245 g**

Lysine content _Terminal ileal digesta_ (g) = 0.86% x 1.97 g / 100% **= 0.017 g**

To determine the amount of lysine released into the small intestine two different methods (direct and indirect) were determined as follows:

TiO_2_ intake (g) = 0.43% x 213 g / 100% **= 0.916 g**

TiO_2_ content _Pooled digesta_ (g) = 1.55% x 31.6 g / 100% **= 0.490 g**

TiO_2_ content _Terminal ileal digesta_ (g) = 4.15% x 1.97 g / 100% **= 0.082 g**

TiO_2_ content _Large intestinal digesta_ (g) = 0.14% x 240 g / 100% **= 0.336 g**

*Direct method*: Lysine released into the large intestine_Direct method_ (g) = 0.336 g x 0.017 g / 0.082 g **= 0.070 g**

Apparent lysine unabsorbed (g) = 0.245 g + 0.017 g + 0.070 g **= 0.332 g**

Apparent lysine disappeared (g) = 6.09 g – 0.332 g **= 5.758 g**

Apparent lysine disappearance (%) = (6.09 g – 0.332 g) / 6.09 g x 100 **= 94.5%**

*Indirect method*: Lysine released into the large intestine_Direct method_ (g) = (0.916 g – (0.490 g + 0.082 g) x 0.017 g / 0.082 g **= 0.072 g**

Apparent lysine unabsorbed (g) = 0.245 g + 0.017 g + 0.072 g **= 0.334 g**

Apparent lysine disappeared (g) = 6.09 g – 0.334 g **= 5.756 g**

Apparent lysine disappearance (%) = (6.09 g – 0.334 g) / 6.09 g x 100 **= 94.5%**

**Supplementary Table 1.** R^2^ and variance values were obtained after fitting different non-linear models to the apparent amino acid absorption data of growing pigs fed a whey protein isolate containing test meal

|  | Asp | |  | Glu | |  | Leu | |
| --- | --- | --- | --- | --- | --- | --- | --- | --- |
|  | R^2^ | Variance |  | R^2^ | Variance |  | R^2^ | Variance |
| Weibull equation | 0.9505 | 232.5 |  | 0.9585 | 190.4 |  | 0.9737 | 153.1 |
| Chapman-Richard’s equation | 0.9735 | 128.2 |  | 0.9735 | 128.2 |  | 0.9813 | 112.2 |
| Logistic function | 0.9739 | 122.6 |  | 0.9754 | 113.0 |  | 0.9813 | 108.9 |
| Gompertz function | 0.9741 | 121.5 |  | 0.9761 | 109.6 |  | 0.9815 | 107.7 |


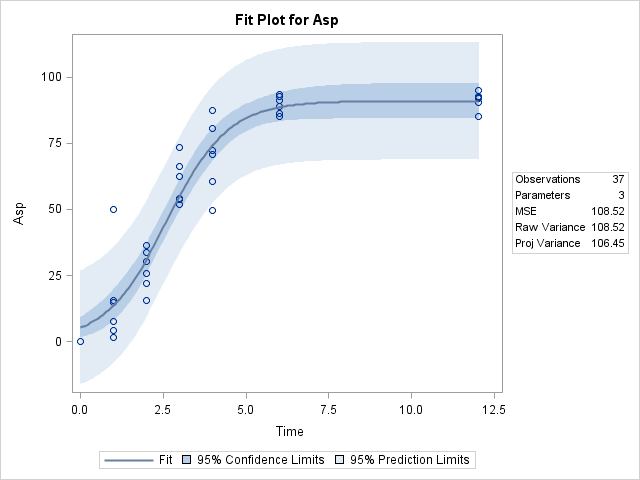


**Supplementary Figure 1.** Observed values and fitted Logistic function for the apparent amino acid absorption of asparagine (Asp) for growing pigs fed a whey protein isolate containing test meal
